# A simple electroelution method for rapid protein purification: isolation and antibody production of alpha toxin from *Clostridium septicum*

**DOI:** 10.7717/peerj.3407

**Published:** 2017-06-22

**Authors:** Lorena Vázquez-Iglesias, Borja Estefanell-Ucha, Leticia Barcia-Castro, María Páez de la Cadena, Paula Álvarez-Chaver, Daniel Ayude-Vázquez, Francisco Javier Rodríguez-Berrocal

**Affiliations:** 1 Department of Biochemistry, Genetics and Immunology, Facultad de Biología, Universidad de Vigo, Vigo, Spain; 2 Unidad de Proteómica, Servicio de Determinación Estructural, Proteómica y Genómica, CACTI, Universidad de Vigo, Spain

**Keywords:** Antibodies, Slot blot, Alpha-toxin, Electroelution, *Clostridium septicum*

## Abstract

*Clostridium septicum* produces a number of diseases in human and farm animals which, in most of the cases, are fatal without clinical intervention. Alpha toxin is an important agent and the unique lethal virulent factor produced by *Clostridium septicum.* This toxin is haemolytic, highly lethal and necrotizing activities but is being used as an antigen to develop animal vaccines. The aim of this study was to isolate the alpha toxin of *Clostridium septicum* and produce highly specific antibodies against it. In this work, we have developed a simple and efficient method for alpha toxin purification, based on electroelution that can be used as a time-saving method for purifying proteins. This technique avoids contamination by other proteins that could appear during other protein purification techniques such chromatography. The highly purified toxin was used to produce polyclonal antibodies. The specificity of the antibodies was tested by western blot and these antibodies can be applied to the quantitative determination of alpha toxin by slot blot.

## Introduction

*Clostridium* is spore forming, Gram-positive and anaerobic (although some species are microaerophilic). Fourteen species that are clearly or potentially pathogenic produce biologically active proteins (toxins), which are responsible for their pathogenicity; some of them are fatal (Hatheway, 1990). *Clostridium* can affect the muscle and subcutaneous tissues of cattle, sheep, goats and other animal species (Rahman et al., 2009).

*Clostridium septicum* is a pathogen which can cause various disease syndromes in animals and humans. However, animal disease syndromes are slightly less understood than in humans (Tweten, 2001). *C. septicum* is the primary etiological agent of a traumatic clostridial myonecrosis, a rapidly fulminating and frequently fatal necrotic disease of the human musculature and was found to be a major cause of gas gangrene due to infection of war wounds (Tweten, 2001). Evidence tends to implicate *C. septicum* as the causative agent of neutropenic enterocolitis (Mirza, McCloud & Cheetham, 2009; Asciutto et al., 2007; King et al., 1984). In animals, wound infections (including castration, docking and partum) are called malignant edema (also known as ‘false’ black quarter, clostridial myonecrosis, and gas gangrene) leading to death within 24 h (Rahman et al., 2009; Songer, 1998). It also produces enteric infections in sheep, known as braxy or bradsot, a fatal bacteremia of the abomasum, causing heavy mortality and important economic loss (reviewed in Songer (1996)).

*C. septicum* produces four extracellular toxins: alpha, beta, gamma and delta (Amimoto et al., 2002; Ballard et al., 1992; Ballard et al., 1995). The alpha toxin is the only known virulence factor of *C. septicum*, a pore-forming toxin that belongs to the aerolysin family of extracellular toxins (Tweten, 2001; Popoff & Bouvet, 2009; Melton-Witt, Bentsen & Tweten, 2006; Diep et al., 1999). The alpha toxin may be closely associated with the disease, and several studies suggest that this toxin may play an important role in the rapidly fatal disease syndromes of *Clostridium septicum.*

*C. septicum* alpha toxin is secreted as an inactive protoxin of 46 kDa that binds to GPI-anchored proteins on the target cell. The bound monomers require proteolytic activation by host proteases, which yield a cytolytically active form that oligomerize into a heptameric complex that inserts into the cellular membrane to form a *β*-barrel pore (Melton et al., 2004). Small pore-forming toxins have a range of effects on the target cell, and alpha toxin is known to have lytic and vacuolating properties (Knapp et al., 2010; Hang’ombe et al., 2004; Wichroski et al., 2002) and is remarkably similar to aerolysin in function (Sellman & Tweten, 1997).

For the detection of *C. septicum* toxins several commercial antibodies have been developed so far, but unfortunately none of them are specific enough to detect only the alpha toxin. Since this toxin is the unique lethal virulence factor produced by *C. septicum*, and due to its industrial interest (Songer, 1996; Cooper, Songer & Uzal, 2013), it is important to develop new and highly specific antibodies that could be used to characterize the protein and design new quantitative methods.

Previous studies have evaluated the isolation of purified alpha-toxin of *C. septicum* and production of polyclonal antibodies against this toxin (Ballard et al., 1992; Vahid et al., 2011). In this study we have developed an efficient, cost-effective and simple method for alpha toxin protein purification based on an electroelution technique. The purified protein was used as immunogen to obtain rabbit polyclonal antibodies. According to our results, the polyclonal antibodies obtained can successfully and specifically detect the alpha toxin of *C. septicum* by western and slot-blot techniques. This approach has not been previously reported for *C. septicum* alpha toxin purification.

## Materials and Methods

### Bacterial strain media and culture conditions

Culture of *C. septicum* was grown anaerobically at optimum temperature for 7 h in specific medium composed of peptones, proteins, glucose and purified water at pH 7.2. Culture supernatant was harvested by centrifuging the cultures at 1,500 g for 30 min at 4 °C.

### Gel electrophoresis and immunoblot procedures

Proteins were separated by sodium dodecyl sulfate-polyacrylamide gel electrophoresis (SDS-PAGE) (Laemmli, 1970) in a 7–10% resolving gel. All samples were diluted into sample buffer that contained 5% *β*-mercaptoethanol and then heated at 95°C for 5 min. Gels were either Coomassie Brilliant Blue R-250 or Oriole™ stained, or blotted. Native-PAGE discontinuous gel electrophoresis was performed in a 6% resolving gel. Gel and reservoir buffer did not contain SDS. The sample was prepared in sample buffer without *β*-mercaptoethanol, SDS or heating. Samples were loaded and an appropriate voltage was stetted (normally 180 V approximately 1 h) to run the electrophoresis.

For immunoblot analysis, proteins that were separated by SDS-PAGE or Native-PAGE were transferred onto polyvinylidene difluoride (PVDF) membranes (Immobilo-P; Merck Millipore, Darmstadt, Germany) according to the manufacturer’s protocols. After transfer, PVDF membranes were blocked with 5% non-fat dry milk in PBS-0.1% Tween 20 (blocking solution). After 60 min, the primary antibodies were diluted in blocking solution and added to the PVDF membrane. After one hour and half incubation at room temperature, the blot was washed three times for 5 min each time with blot wash buffer (PBS-0.1% Tween 20) to remove unbound antibody. The blot was then incubated for 45 min with the appropriate secondary conjugate antibody (Anti-Rabbit IgG (whole molecule)-Alkaline Phosphatase-A3812; Sigma-Aldrich, St. Louis, MO, USA) diluted 1:15,000 in blocking solution. The bands recognized by the primary antibody were visualized by using a BCIP/NBT substrate (Roche Pharma, Madrid, Spain), according to manufacturer’s instructions, for 5 min to allow colour development. Blots were digitized with a calibrated densitometer (GS-800; Bio-Rad, Madrid, Spain) and images were analyzed with Quantity One 4.4.1 software (Bio-Rad, Madrid, Spain).

### Identification of bands by mass spectrometry

For identification, protein bands were cut out from Oriole™ stained gels and destained sequentially with ammonium bicarbonate (AmBic) 25 mM and 50% ACN/AmBic 25 mM. Proteins were reduced by treatment with 10 mM DTT (dithiothreitol) for 1 h at 56 °C, and alkylated with 55 mM IAA (iodoacetamida) for 30 min at room temperature. Samples were dried and digested with 40 ng of modified porcine trypsin (Promega Biotech, Madrid, Spain) in 25 mM AmBic at 37 °C overnight. After digestion, peptides were eluted first with 0.5% (v/v) TFA for 30 min at room temperature and second with 100% (v/v) ACN (acetonitrile) for 15 min at 37 °C. Finally 3 µL of digests were applied onto a disposable Anchorchip™ MTP-sized MALDI target prespotted with HCCA (*α*-cyano-4-hydroxycinnamic acid) matrix (Bruker Daltonics, Madrid, Spain), and analyzed by matrix-assisted laser desorption/ionization-time of flight tandem mass spectrometry (MALDI-TOF/TOF) with an Autoflex III smartbeam (Bruker Daltonics, Madrid, Spain) in positive ion reflector mode. Samples were also analyzed by high-performance liquid-chromatography (HPLC), with an UltiMate3000 (Dionex), coupled to a FTICR Apex-Qe (Bruker Daltonics, Madrid, Spain) to perform nano-electrospray fourier transform ion cyclotron resonance mass spectrometry (nESI-FTICR). Data were generated in PKL and mgf file formats with FlexAnalysis 3.0 and DataAnalysis 4.0 softwares (Bruker Daltonics, Madrid, Spain), for MALDI-TOF/TOF and HPLC-nESI-FTICR methods respectively, and then submitted for database searching against SwissProt 56.6 (405506 sequences; 146166984 residues) and NCBInr 20070216 (4626804 sequences; 1596079197 residues) databases by BioTools 3.0 software (Bruker Daltonics, Madrid, Spain) through the version 2.2.04 of MASCOT search engine (Matrix Science, London, UK). Search parameters were set as follows: taxonomy Bacteria/Firmicutes; enzyme trypsin; allowance of one missed cleavage site; carbamidomethyl of cysteine as fixed modification; oxidation of methionine as variable modification; monoisotopic mass values; 100 ppm (MALDI) or 6 ppm (ESI) of mass tolerance for precursor ions; 0.5 Da (MALDI) or 0.01 Da (ESI) of mass tolerance for fragment ions and protein mass unrestricted.

### Electroelution device and procedure

After separation by SDS-PAGE in four 7% resolving gels of 1.5 mm, the band representing the high-molecular-weight complex was excised from the gel using a sharp disposable blade and subjected to electroelution in the presence of SDS by using an electroeluter (Model 422; Bio-Rad, Madrid, Spain). Proteins were electroeluted for 3 h at room temperature using elution buffer (25 mM Tris–HCl, pH 8.3, 192 mM glycine, containing 0.1% (w/v) SDS), following the manufacturer’s instructions. In order to remove SDS from the preparation, the elution procedure was continued for another 2 h using fresh elution buffer without SDS. After that, the protein was recovered in a minimal amount of the same buffer and concentrated using an Amicon®Ultra-15 (Ultracel®-10K; Merck Millipore, Darmstadt, Germany). The purity of protein was checked by SDS–PAGE and the presence of protein complexes was analyzed by 7% SDS-PAGE. The identity of the purified protein was confirmed by mass spectrometry.

### Protein estimation

The concentration of soluble proteins was determined according to the method of Bradford (1976).The optical density was read at 595 nm and the amount of protein was calculated by using BSA (Sigma Aldrich, Madrid, Spain) as standard.

### Rabbit polyclonal antibody production

Three New Zealand White rabbits were injected five times subcutaneously with 100 μg of purified alpha toxin oligomer of *C. septicum* and a water soluble adjuvant (incomplete Freund’s adjuvant). The first dose was double following a co-injection protocol (two consecutive injections, one on each side of the belly). The third, fourth and fifth injections were performed every two weeks. Two months after the initial immunization the animals, properly anesthetized with ketamine plus xylacina, were bled by cardiac puncture. The blood was allowed to clot for 24 h at 4 °C, and the serum was collected after centrifugation. Euthanasia of anesthetized animals was for cerebral concussion (blunt blow to the head). Before removing the bodies, cessation of vital signs and appearance of rigor mortis was tested. Daily monitoring protocol per animal was covered, using the proposed model by Morton & Griffiths (1985). The supervision protocol establishes recommendations of analgesia and endpoint application. It can be said that it was not necessary in any case because no animal got a score > 5 monitoring protocols. Although endpoint criteria were established, it was not necessary to apply.

All experiments were performed under the guidelines of the European Community for animal use for scientific purpose (2010/60/UE). The handling procedures and sampling frequency were designed to reduce stress and health risks for subjects according to Spanish laws (RD53/2013). Favourable report from the Ethics Committee of Animal Welfare was obtained and was notified as required by current legislation (RD 1201/2005). The antibody was produced by CZ Veterinaria S.A. (36400 Porriño-Pontevedra; Spain).

Immunoblot analysis were made for evaluation of antibody specificity Sample of protein purified and indentified as alpha toxin of *C. septicum* was used for this assay and was consider a positive control.

### Slot blot immunoassay

For slot blotting, 0.2 mL of each sample was applied to a slot blot manifold Bio-Dot SF 48 well slot format apparatus (Bio-Rad, Madrid, Spain) and drawn by vacuum through a pre-wetted polyvinylidene fluoride (PVDF) membrane. Unbound membrane sites were blocked for 1 h with 5% non-fat dry milk in PBS containing 0.1% Tween-20 (blocking solution), and the membrane was then incubated 90 min at room temperature with a 1:10,000 dilution of rabbit anti-*C. septicum* alpha toxin antiserum diluted in blocking solution. The membrane was washed three times with blot wash buffer (PBS-0.1% Tween 20), and incubated 45 min at room temperature with a 1:15,000 dilution of alkaline phosphatase-conjugated goat anti-rabbit immunoglobulin G (Sigma Aldrich, Madrid, Spain) diluted in blot wash buffer. Again, the membrane was washed three times with blot wash buffer. Bands were visualized using BCIP/NBT (Roche Pharma, Madrid, Spain), according to the manufacturer’s instructions. Blots were digitized with a calibrated densitometer (GS-800; Bio-Rad, Madrid, Spain) and the intensity of each band on the slot blot was measured using Quantity One 4.4.1 software (Bio-Rad, Madrid, Spain). A four-parameter logistic function (4PL) was used for calculating the alpha toxin concentration (Wild, 2013) using the MaterPlex Reader Fit software (MiraiBio Group; Analysis Software for the Life Sciences; Hitachi Solutions America, Ltd., San Bruno, CA, USA).

## Results

### Identification of *Clostridium septicum* alpha toxin protein

**Figure 1:**
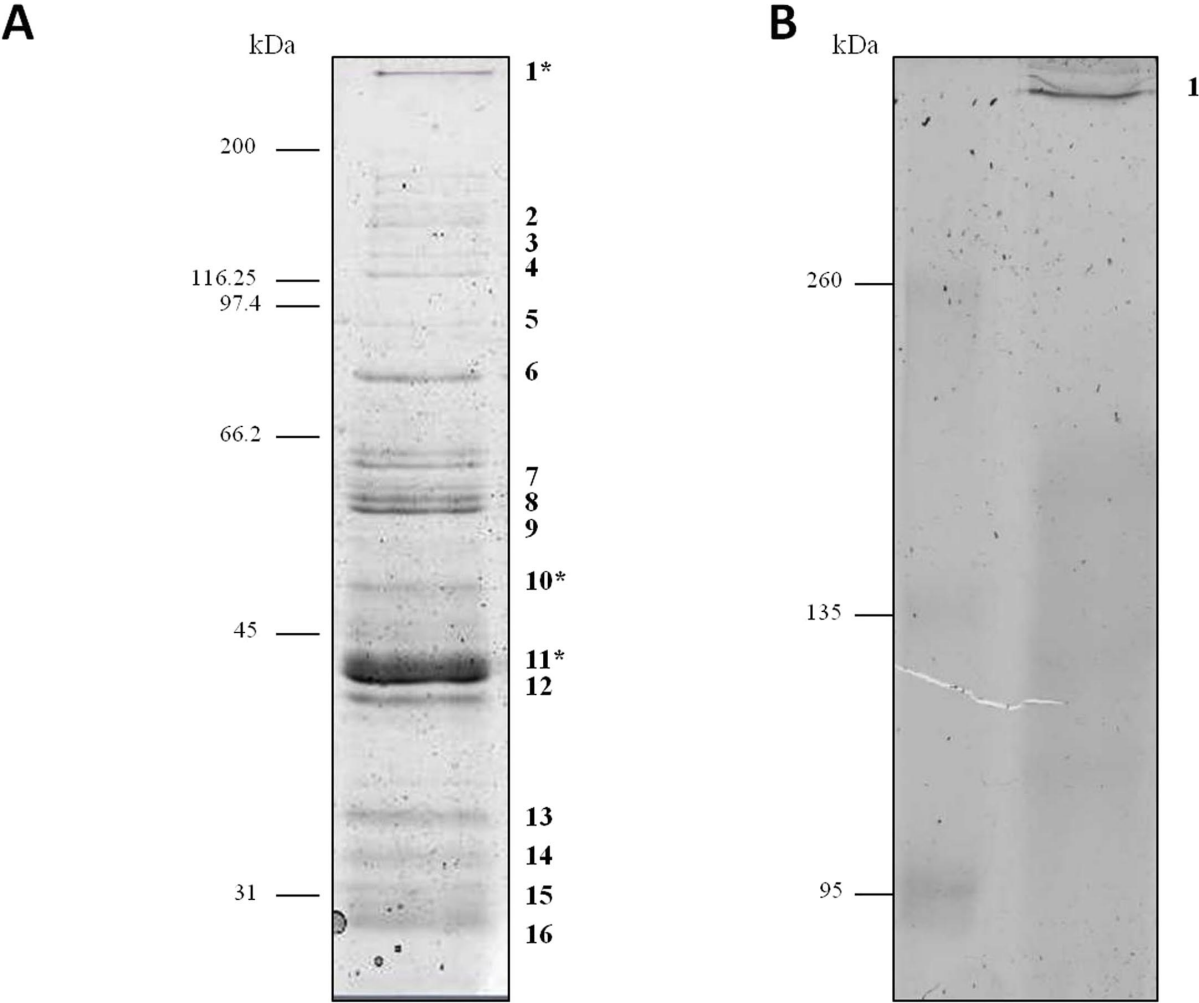
Protein profile of *Clostridium septicum* extracellular medium. (A) SDS-PAGE analysis by Coomassie Brilliant Blue R-250 stained. (B) Non-denaturing Native-PAGE analysis by Coomassie Brilliant Blue R-250 stained. Protein bands 1–17 were cut and analyzed by MS/MS. kDa indicates migration of the protein markers.

Proteins secreted into extracellular medium by *C. septicum* were separated according to their molecular mass by SDS-PAGE as described above (Fig. 1). The most abundant bands were cut off the gel and submitted to mass spectrometry analysis for protein identification. The identity of proteins achieved either by MALDI-TOF/TOF or HPLC-nESI-FTICR is summarized in Table 1. The protein profile analyses of samples revealed the presence of different proteins, all of them belonging to *C. septicum*. Three of them were identified as *C. septicum* alpha toxin: a high molecular weight protein (greater than 200 kDa) and two proteins of about 49 kDa and 43 kDa (see Fig. 1A: band 1, 10 and 11, respectively).

**Table 1:** List of protein identified by MALDI-TOF/TOF or HPLC-nESI-FTICR in *Clostridium septicum* extracellular medium.

Band^a^	M_*r*_^b^ (kDa)	MS method^c^	Protein name	SwissProt/NCBInr accession number	Function
1, 17	>200	MALDI	Alpha toxin	Q53482/452163/89257968	Pathogenesis
2	165.3	ESI	Hypothetical protein CPE1875	Q8XJ86/18310857	–
3	128.8	ESI	Pyruvate-flavodoxin oxidoreductase	WP_011969176.1	Oxidoreduction
4	111.1	MALDI/ESI	Collagenase	Q84IM7/28557072	Digestion of collagen
5	83.3	ESI	Formate acetyltransferase	Q8XL89/18310135	Glucose metabolic process
6	83.3	ESI	Formate acetyltransferase	Q8XL89/18310135	Glucose metabolic process
7	60	ESI	60 kDa chaperonin	Q0PVX4/110555138	Chaperone
8	56.7	MALDI/ESI	2,3-bisphosphoglycerate-independent phosphoglycerate mutase	Q0STD7/110803633	Catalyzes interconversion of 2-phosphoglycerate and 3-phosphoglycerate
9	60.3	ESI	Formate-tetrahydrofolate ligase	Q8XHL4/489554783	Tetrahydrofolate interconversion
10	49.7	MALDI/ESI	Alpha toxin Glucose-6-phosphate isomerase	Q53482/452163 Q8XI54/489557801	Pathogenesis; Carbohydrate degradation & glycolysis
11	49.9	MALDI/ESI	Alpha toxin	Q53482/452163	Pathogenesis
	42.0		Flagellin protein FliA(S)	Q8RRA0/19910961	Ciliary or flagellar motility
	41.0		Thiolase	82747493	Acyltransferase
12	35.5	ESI	Hypothetical protein CPE1232	Q8XL10/18310214	–
	38.1		UDP-4-dehydro-6-deoxy-2-acetamido- D-glucose 4-reductase	Q893U5/28211366	Lyase and oxidoreductase activities;
	42.0		Flagellin protein FliA(S)	Q8RRA0/19910961	Ciliary or flagellar motility;
	41.0		Thiolase	82747493	Acyltransferase
13	30.5	MALDI/ESI	3-hydroxybutyryl-coA dehydrogenase	A0Q2T9/118443750	Oxidoreductase
14	30.5	ESI	3-hydroxybutyryl-coA dehydrogenase	A0Q2T9/118443750	Oxidoreductase
15	25.1	ESI	Hypothetical protein CPE1233	Q8XL09/18310215	–
	30.4		Fructose-bisphosphate aldolase	Q97KT9/15894114	Glycolysis
16		MALDI/ESI	Triosephosphate isomerase	Q64G20/52082975	Isomerase
			Hypothetical protein CPE1233	Q8XL09/18310215	–

**Notes.**

a Band number as stated in Fig. 1.

b Theoretical molecular mass (kDa) taken from the UniProtkB entry.

c Type of ionization used for mass spectrometry.

We also analyzed the protein profile of *C. septicum* secreted proteins by Native-PAGE (6% resolving gel), in order to test the alpha toxin in its native state. As shown in Fig. 1B, after Coomassie Brilliant Blue R-250 stain, a clear band greater than 260 kDa was observed (band 17). This band was identified by mass spectrometry as *C. septicum* alpha toxin and we concluded that corresponds to its oligomeric form. The few protein bands observed between 260 and 95 kDa molecular weight range (see Fig. 1A) appeared as a faint smear in native conditions (Fig. 1B).

### Purification of alpha toxin oligomer for polyclonal antibodies production

For antibody production, the band corresponding to the alpha toxin oligomeric form (band 1) was excised from the gel after resolving secreted proteins of *C. septicum* by SDS-PAGE. Protein purification was carried out through the active elution from gel pieces using the electroelution device as described in ‘Material and Methods’ section. Analytical electrophoresis of the electroeluted protein was performed in order to confirm the purity of the alpha toxin (Fig. S1). After electroelution the purified alpha toxin was concentrated by Amicon®Ultra-15, yielding approximately 2.5 mg of total alpha toxin. About 0.5 mg of purified protein was injected into each rabbit and after two months the animals were bled.

### Evaluation of the *Clostridium septicum* alpha toxin polyclonal antibodies by western-blot technique

**Figure 2:**
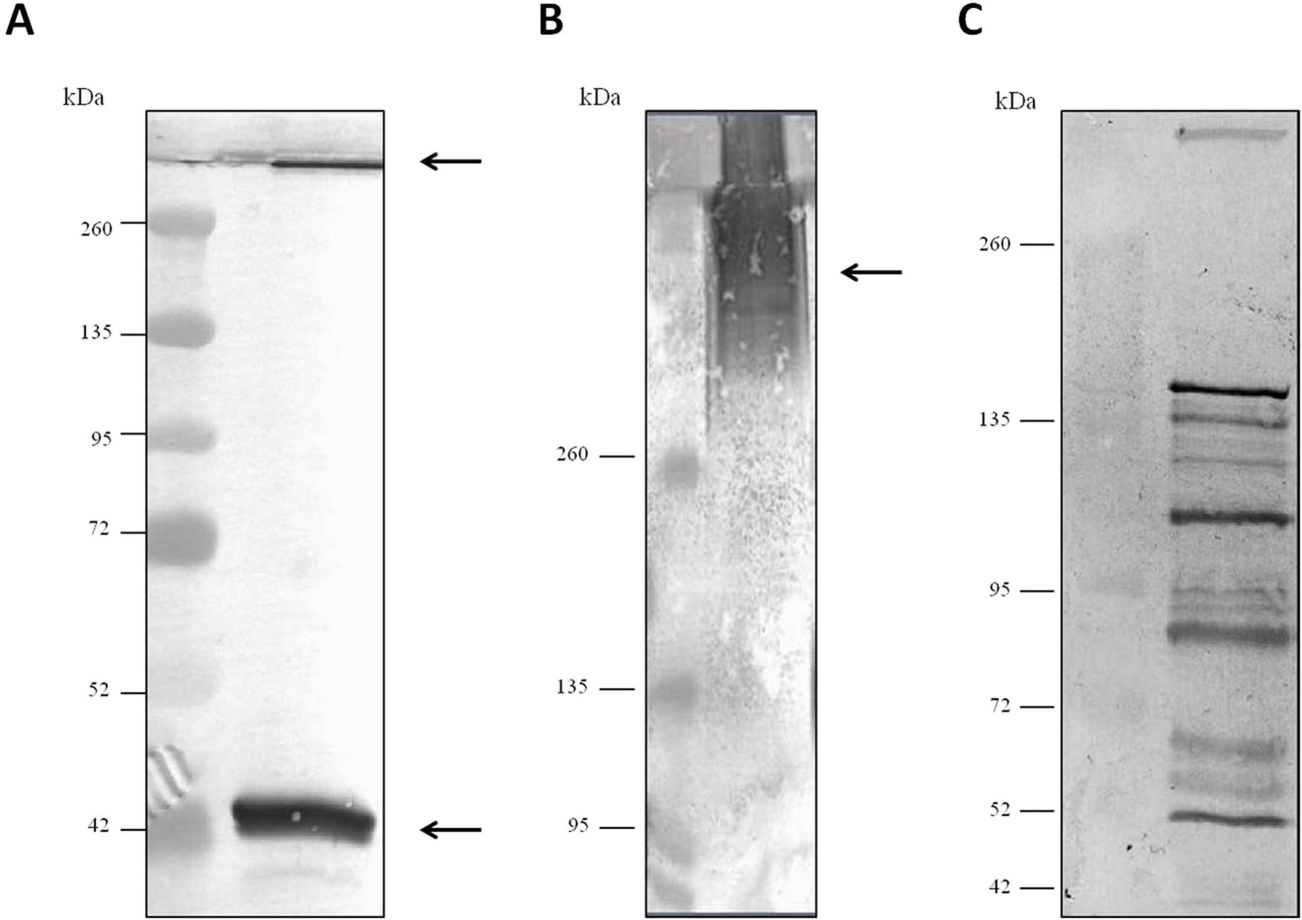
Western blot analysis of the *Clostridium septicum* extracellular medium using our anti-*Clostridium septicum* alpha toxin and the *Clostridium septicum* (Gas-Gangrene) antitoxin (NIBSC code: VI). (A) SDS-PAGE and western blot analysis using anti-*C. septicum* alpha toxin. (B) Native-PAGE and western blot analysis using anti-*C. septicum* alpha toxin. (C) SDS-PAGE and western blot analysis using the *C. septicum* (Gas-Gangrene) antitoxin (NIBSC code: VI). kDa indicates migration of the protein markers. The arrows indicate alpha toxin. A total of 3 μg of total protein per lane were loaded.

**Figure 3:**
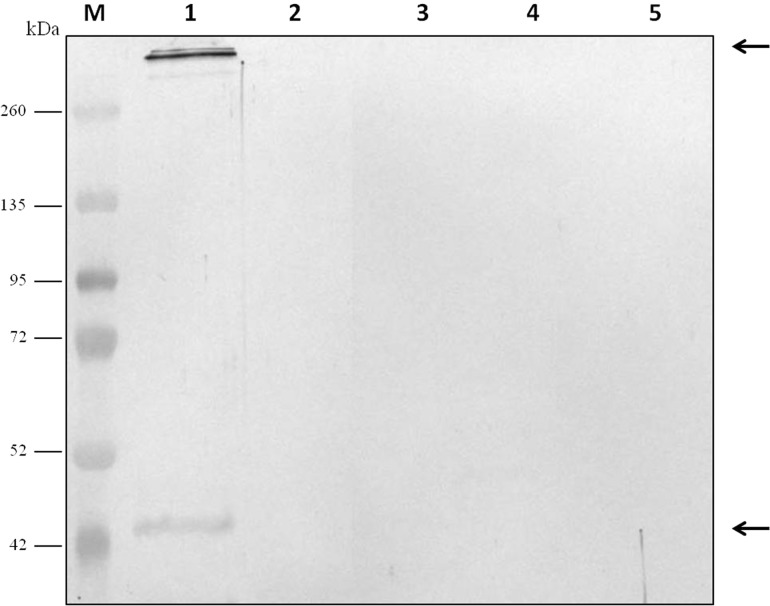
Analysis of the specificity of the anti-*Clostridium septicum* alpha toxin by western blotting with other *Clostridium spp.* Lane 1 extracellular medium of *C. septicum* Lane 2 extracellular medium of *C. perfringens* type D, Lane 3 extracellular medium of *C. haemolyticum,* Lane 4 extracellular medium of *C. tetani* and Lane 5 extracellular medium of *C. sordellii.* Lane M indicates migration of protein molecular weight markers (kDa)*.* The arrows indicate alpha toxin. A total of 3 μg of total protein per lane were loaded.

For evaluation of antibody specificity, the anti-*C. septicum* alpha toxin rabbit antiserum was subjected to immunoblot analyses. Samples of proteins secreted by *C. septicum* culture were used for these assays. As shown in Fig. 2A, the antiserum detected two bands of approximately 43 kDa and a higher band greater than 200 kDa. We also tested the antiserum under native conditions, detecting a strong smear over 200 kDa (Fig. 2B). In addition, we also evaluated the result obtained with the *C. septicum* antitoxin antiserum purchased from NIBSC and we could prove that this second antibody recognized a lot of non-specific proteins (Fig. 2C). Finally, we assessed the specificity of our antibody regarding other *Clostridium spp*. extracellular culture samples: *C. perfringens* type D*, C. haemolyticum, C. tetani* and *C. sordellii* and interestingly no reaction was observed (Fig. 3).

### Evaluation of the *Clostridium septicum* alpha toxin polyclonal antibodies by slot-blot technique

**Figure 4:**
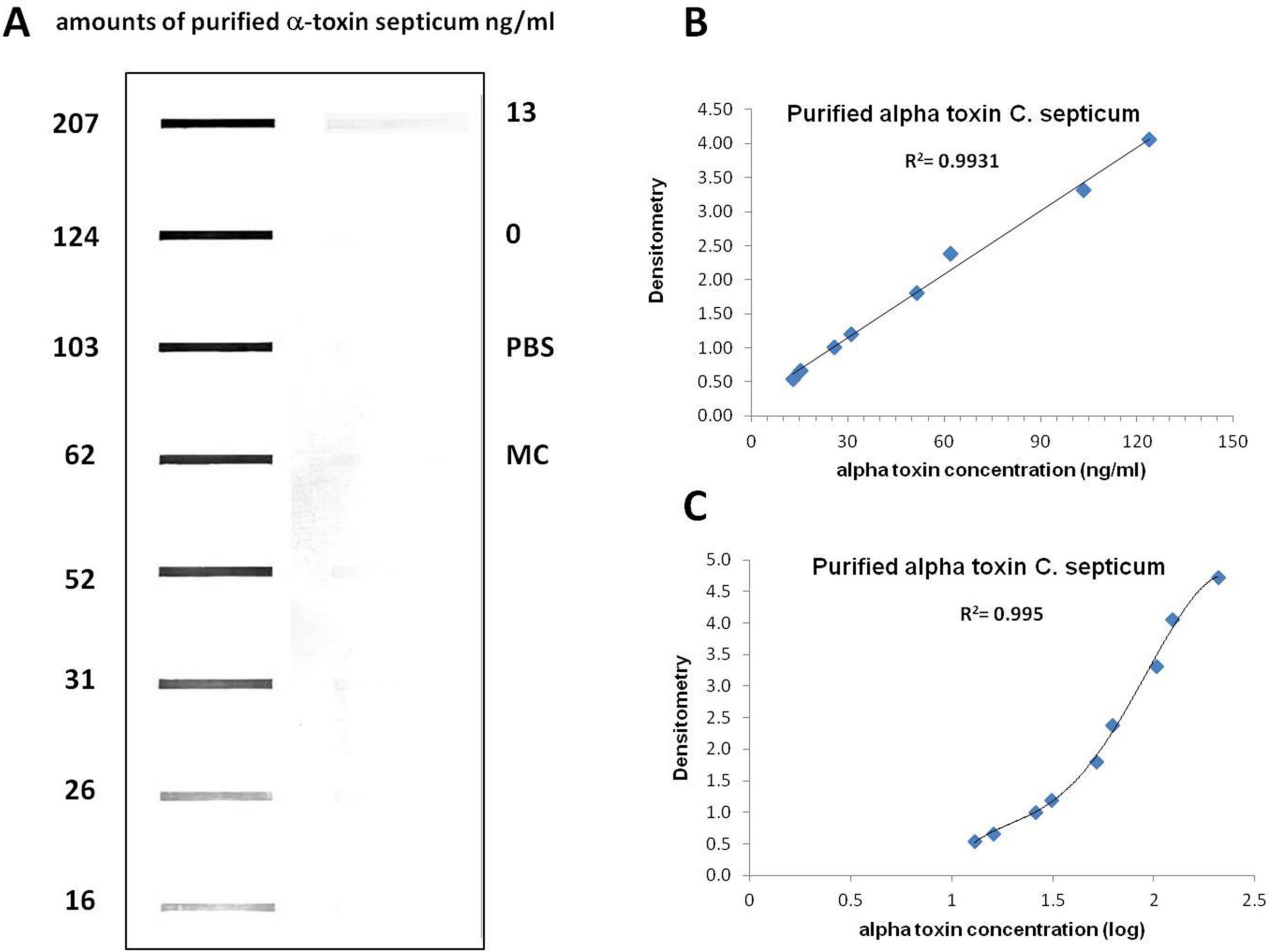
Slot blot analysis for the quantification of different concentrations of purified alpha toxin. (A) Several concentrations of purified alpha toxin were analyzed using the protocol detail in ‘Material and Methods’. The culture medium and PBS serve as a negative control. (B) This figure shows the linear relationship between optical density and protein concentration gave a correlation coefficient of 0.9931. (C) The logarithm concentration of the alpha toxin was plotted on *x* axis and the O.D. values were plotted in *y* axis. The 4-parameter logistic (4PL) model was used for the calibration curve. A very good fitting, *R*^2^ = 0.995, was demonstrated.

In order to evaluate the efficacy of the antibody to recognize the purified toxin, we used the slot-blot technique. The oligomeric form of alpha toxin was purified as mentioned in ‘Materials and Methods’ and concentrated up to 62 µg/mL, which was used as starting solution to generate protein loadings ranging from 13 ng/mL to 207 ng/mL. As shown in Fig. 4A, the intensities of the blot decrease proportionally with decreasing concentrations of alpha toxin. Figure 4B shows that the concentration of the antigen plotted on *x* axis is proportional to the OD values (*y* axis). A linear relationship between optical density and protein concentration was verified by linear regression analyses, which gave a correlation coefficient of 0.9931 (from 13 ng/mL to 124 ng/mL). The 4-parameter logistic (4PL) model was used for the calibration curve fitting. The curve demonstrates a very good fitting, as revealed by *R*^2^ = 0.995, with all concentrations of purified alpha toxin assayed (Fig. 4C). We also tested the specificity of this antibody with the culture medium and PBS and no specific binding was detected (Fig. 4A).

## Discussion

For studies related with *C. septicum* alpha toxin*,* either for research or in industry, the WHO International Standard *C. septicum* Antitoxin purchased from the NIBSC is used worldwide. We could corroborate by western blot that this antiserum recognized different proteins in *C. septicum* cultures, besides alpha toxin. Therefore, in the present work, we provided a purification method of *C. septicum* alpha toxin that allowed us to obtain highly specific antibodies against this antigen.

First, we resolved culture samples by SDS-PAGE and subsequently the proteins were detected with Oriole™ staining. We chose Oriole fluorescent gel stain because it consists in a one-step room temperature staining protocol (without prior fixing or subsequent destaining steps) and is fully compatible with downstream mass spectrometry analysis (Thulasiraman & Waker, 2010). Mass spectrometry allowed us to identify *C. septicum* alpha toxin in three of the visualized bands. The most intense band is about 43 kDa and corresponds to the monomer of the alpha toxin, in agreement with a previous study where alpha toxin was purified as a basic protein of approximately 48 kDa (Ballard et al., 1992). We have also identified the oligomeric form (SDS and *β*-mercaptoethanol resistant) with a molecular weight greater than 200 kDa and a faint protein band of about 49 kDa. Ballard et al. (1992) described two different proteins that were immunologically related to alpha toxin that were always present in purified preparations. One of these proteins was approximately 4–5 kDa smaller than the alpha toxin and the other one was six or seven times greater than alpha toxin. These data are in perfect agreement with our results.

In order to do further biological studies, we decided to purify alpha toxin. We chose its oligomeric form because the subunit interactions within a multimeric protein are generally retained (Janin, Bahadur & Chakrabarti, 2008; Nooren & Thornton, 2003). Purification was achieved using an electroelution method that allowed us a rapid and quantitative elution of proteins from denaturing gels. This method can be run as a preparative or an analytical mode since both yield biologically active material, such as proteins (Seelert & Krause, 2008), nucleic acids (Symons & Clarkson, 1988) and oligosaccharides (Al-Hakim & Linhardt, 1990). The only prerequisite for electroelution is that the molecule of interest can move in an electric field (Persidis & Harcombe, 1992). It is an efficient and reproducible method that could be used as a routine method for eluting multiple protein bands, allowing considerable time saving. It is simple, fast, and cost-effective; it does not need an expensive apparatus and reagents, and does not require prior knowledge of the protein characteristics. This technique is applicable not only to soluble proteins but also to membrane-bound proteins and it is applicable to raise antibodies as well (Ohhashi et al., 1991). In addition, purification of proteins, particularly toxins, based on electroelution has been successfully implemented in previous studies (Borowiec et al., 2016; Hewlett et al., 1989). The small recovery volume makes the samples easy to handle, and several samples should be eluted simultaneously to yield large amounts of protein. As other authors (Ohhashi et al., 1991), we could readily obtain milligram amounts of proteins with this method, enough to raise antibodies.

Once the polyclonal antibodies against *C.septicum* alpha toxin were obtained, the western blot technique was used to examine the specificity of the antiserum in different samples. We concluded that this antibody only recognizes two different forms of alpha toxin (monomer and oligomer) in culture samples under denaturing conditions and the oligomer form in native gels. It is important that the antibody can recognize alpha toxin in a native state in order to be used in the future in techniques such as slot blot. In immunoquantitative methods, proteins are in native state so alpha toxin would be in the oligomeric form.

Besides, we could prove that our antiserum does not recognize any proteins in cultures of *C. perfringens* type D*, C. haemolyticum, C. tetani* and *C. sordellii*.

Because of the fact that we had obtained a very specific antibody, the next step was to prove if it could be used to detect and quantify *C. septicum* alpha toxin. For this purpose, we used the immunoquantitative slot blot technique, confirming that this antibody was able to recognize small amounts of purified alpha toxin. This method is simple, faster and more sensitive than the western blot and due to its high sensitivity, accuracy and ease of use, this assay can be comparable with other immunoassays such as ELISA (Zhu, Saul & Miles, 2005; Li et al., 2010). Due to these advantages, this assay was previously reported for the detection and quantification of other toxins as *Clostridium botulinum* neurotoxin (Cadieux et al., 2005) or yessotoxins produced by dinoflagellates (Pierotti et al., 2007). In addition, this technique could be used for specific detection of native multi-subunit proteins (Burke & Barik, 1998) and allows the detection of the expression of toxins directly from culture supernatants without any previous treatment (Vilhena-Costa et al., 2006; Bellini et al., 2005; Navarro-García et al., 1998).

In conclusion here we show that identification of proteins by mass spectrometry after SDS-PAGE separation coupled to the electroelution technique is an easy, rapid and cost-effective technique to purify the alpha toxin from *C. septicum.* Besides, we were able to obtain a specific antibody against the *C. septicum* alpha toxin that can be used in the western blot and slot blot assays. This methodology could serve to purify other types of toxins and proteins.

##  Supplemental Information

10.7717/peerj.3407/supp-1 Figure S1 Analytical electrophoresis of the electroeluted protein. Purity of the electroeluted protein was checked by Coomassie Brilliant Blue R-250 stained. (A) Protein composition of electroeluted protein that migrated as an oligomeric form. (B)Protein composition of electroeluted protein that migrated as an oligomeric form and as a monomeric form. kDa indicates migration of the protein markers.

10.7717/peerj.3407/supp-2 Supplemental Information 2 Supplemental File 1 Mascot Search Results of mass spectrometry identification for bands 1 and 17 (band 17 is renamed band 4).

10.7717/peerj.3407/supp-3 Supplemental Information 3 Supplemental File 2 Mascot Search Results of mass spectrometry identification for purified electroeluted protein.

## Additional Information and Declarations

### Data Availability

The following information was supplied regarding data availability: The raw data has been uploaded as Supplemental Files.
