# Supplementary figures and images for: A simple electroelution method for rapid protein purification: isolation and antibody production of alpha toxin from Clostridium septicum
Source: PeerJ. 2017 Jun 22;5:e3407. doi: 10.7717/peerj.3407 (PMC5483040; doi:10.7717/peerj.3407)

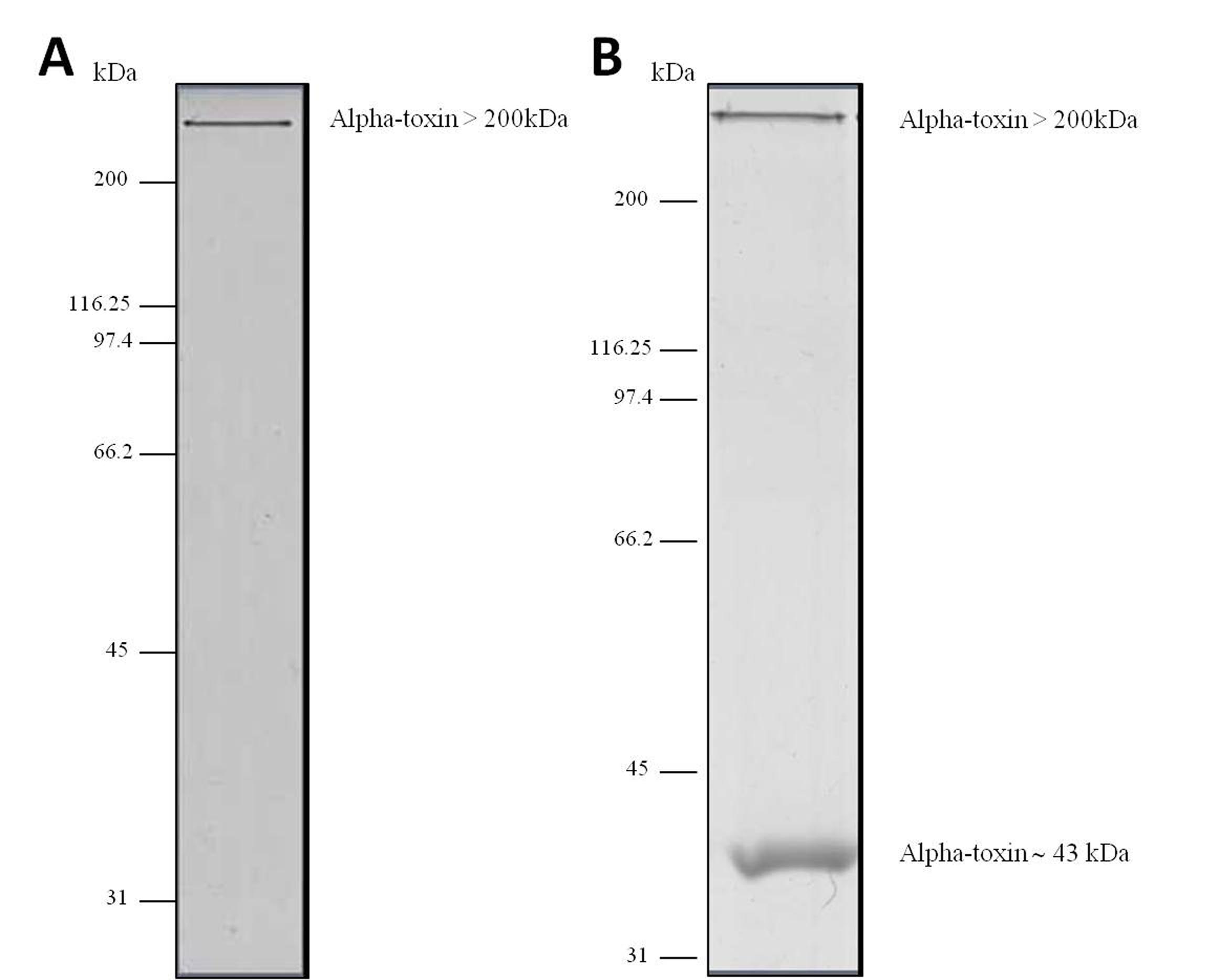

Supplement: Figure S1 — Purity of the electroeluted protein was checked by Coomassie Brilliant Blue R-250 stained. (A) Protein composition of electroeluted protein that migrated as an oligomeric form. (B)Protein composition of electroeluted protein that migrated as an oligomeric form and as a monomeric form. kDa indicates migration of the protein markers. [file peerj-05-3407-s001.jpg]
