# Supplementary material for: A simple electroelution method for rapid protein purification: isolation and antibody production of alpha toxin from Clostridium septicum
Source: PeerJ. 2017 Jun 22;5:e3407. doi: 10.7717/peerj.3407 (PMC5483040; doi:10.7717/peerj.3407)
Supplement: Supplemental Information 2 — Mascot Search Results of mass spectrometry identification for bands 1 and 17 (band 17 is renamed band 4). [file peerj-05-3407-s002.pdf]

Supplemental File 1: Mascot Search Results of mass spectrometry identification for bands 1 and 17 (band 17 is rename band 4).

Mascot Search Results

```
User            : Unidad Proteomica CACTI
Email           : proteomica@uvigo.es
Search title    : Banda 1 MSMS
MS data file    : DATA.TXT
Database        : NCBInr 20070216 (4626804 sequences; 1596079197 residues)
Taxonomy        : Firmicutes (gram-positive bacteria) (320691 sequences)
Timestamp       : 24 Feb 2011 at 15:37:44 GMT
Protein hits    : gi|452163    alpha-toxin [Clostridium septicum]
                  gi|89099867 Aminopeptidase N [Bacillus sp. NRRL B-14911]
                  gi|77683548 conserved hypothetical protein [Alkaliphilus metalliredigenes QYMF]
                  gi|30023346 uracil phosphoribosyltransferase [Bacillus cereus ATCC 14579]
                  gi|15616326 uracil phosphoribosyltransferase [Bacillus halodurans C-125]
```

	NCBInr	Decoy	False discovery rate
Peptide matches above identity threshold	1	0	0.00 %
Peptide matches above homology or identity threshold	2	0	0.00 %

Mascot Score Histogram

Ions score is -10*Log(P), where P is the probability that the observed match is a random event.
Individual ions scores > 38 indicate identity or extensive homology (p<0.05).
Protein scores are derived from ions scores as a non-probabilistic basis for ranking protein hits.

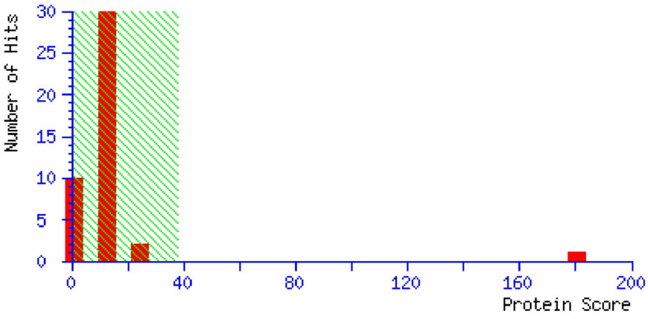

Peptide Summary Report

Format As | Peptide Summary | Help

Significance threshold p< 0.05 | Max. number of hits 5

Standard scoring ◉ MudPIT scoring ○ | Ions score or expect cut-off 0 | Show sub-sets 0

Show pop-ups ◉ Suppress pop-ups ○ | Sort unassigned Decreasing Score | Require bold red ☐

Overview Table

Click on column header to jump to entry in results list.
Move mouse over any indicator to highlight identical peptides.
Click on an indicator to see details of individual match.
Use check boxes to select sub-set of queries for new search.

Mouse over: -Query-
-Accession- -Sequence-

Hit:	1	2	3	4	5
☑ 908.52 (1+)	●	●		●	●
☑ 1026.48 (1+)	●				
☑ 1130.62 (1+)	●		●		
☑ 1326.63 (1+)	●				

☑ 1416.63 (1+)

Select All | Select None | Search Selected | ☐ Error tolerant | Archive Report

1. gi|452163 **Mass:** 49926 **Score:** 180 **Matches:** 5(1) **Sequences:** 5(1)
alpha-toxin [Clostridium septicum]
☐ Check to include this hit in error tolerant search or archive report

Query	Observed	Mr(expt)	Mr(calc)	ppm	Miss	Score	Expect	Rank	Unique	Peptide
1	908.52	907.51	907.51	1.13	0	27	0.93	1	U	K.VSYLLSAR.Y
2	1026.48	1025.48	1025.48	-0.04	0	29	0.43	1	U	K.NSYDALTSR.K
3	1130.62	1129.61	1129.61	0.51	0	38	0.081	1	U	K.IIQDPEFIR.N
4	1326.63	1325.63	1325.62	4.20	0	26	0.9	1	U	R.EDHTEDRPTVK.L
5	1416.63	1415.62	1415.61	6.47	0	61	0.00025	1	U	R.YNPNDPYASGYR.A

Proteins matching the same set of peptides:
gi|27531076 **Mass:** 49634 **Score:** 180 **Matches:** 5(1) **Sequences:** 5(1)
alpha-toxin [Clostridium septicum]
gi|27531078 **Mass:** 49650 **Score:** 180 **Matches:** 5(1) **Sequences:** 5(1)
alpha-toxin [Clostridium septicum]
gi|27531080 **Mass:** 49588 **Score:** 180 **Matches:** 5(1) **Sequences:** 5(1)
alpha-toxin [Clostridium septicum]
gi|89257968 **Mass:** 49513 **Score:** 180 **Matches:** 5(1) **Sequences:** 5(1)
alpha-toxin [Clostridium septicum]

---

2. gi|89099867 **Mass:** 91440 **Score:** 25 **Matches:** 1(0) **Sequences:** 1(0)
Aminopeptidase N [Bacillus sp. NRRL B-14911]
☐ Check to include this hit in error tolerant search or archive report

Query	Observed	Mr(expt)	Mr(calc)	ppm	Miss	Score	Expect	Rank	Unique	Peptide
1	908.52	907.51	907.48	41.2	0	25	1.5	2	U	K.SVYIEAAR.A

---

3. gi|77683548 **Score:** 20 **Matches:** 1(0) **Sequences:** 1(0)
conserved hypothetical protein [Alkaliphilus metalliredigenes QYMF]
☐ Check to include this hit in error tolerant search or archive report

Query	Observed	Mr(expt)	Mr(calc)	ppm	Miss	Score	Expect	Rank	Unique	Peptide
3	1130.62	1129.61	1129.61	4.09	1	20	4.8	2	U	R.LIRSEEDLR.D

---

4. gi|30023346 **Mass:** 22943 **Score:** 18 **Matches:** 1(0) **Sequences:** 1(0)
uracil phosphoribosyltransferase [Bacillus cereus ATCC 14579]
☐ Check to include this hit in error tolerant search or archive report

Query	Observed	Mr(expt)	Mr(calc)	ppm	Miss	Score	Expect	Rank	Unique	Peptide
1	908.52	907.51	907.51	1.13	1	18	8.3	3	U	K.ITYIRDK.N

Proteins matching the same set of peptides:
gi|47570682 **Mass:** 8607 **Score:** 18 **Matches:** 1(0) **Sequences:** 1(0)
uracil phosphoribosyltransferase [Bacillus cereus G9241]
gi|89203556 **Score:** 18 **Matches:** 1(0) **Sequences:** 1(0)
gi|89207647 **Score:** 18 **Matches:** 1(0) **Sequences:** 1(0)
gi|118480351 **Score:** 18 **Matches:** 1(0) **Sequences:** 1(0)

---

5. gi|15616326 **Score:** 17 **Matches:** 1(0) **Sequences:** 1(0)
uracil phosphoribosyltransferase [Bacillus halodurans C-125]
☐ Check to include this hit in error tolerant search or archive report

Query	Observed	Mr(expt)	Mr(calc)	ppm	Miss	Score	Expect	Rank	Unique	Peptide
1	908.52	907.51	907.51	1.13	1	17	9.1	4	U	K.LTYIRDK.S

Proteins matching the same set of peptides:

gi|22096373	**Score:** 17	**Matches:** 1(0)	**Sequences:** 1(0)
gi|23100439	**Score:** 17	**Matches:** 1(0)	**Sequences:** 1(0)
gi|56421903	**Score:** 17	**Matches:** 1(0)	**Sequences:** 1(0)

Search Parameters

Type of search : MS/MS Ion Search
Enzyme : Trypsin
Fixed modifications : Carbamidomethyl (C)
Variable modifications : Oxidation (M)
Mass values : Monoisotopic
Protein Mass : Unrestricted
Peptide Mass Tolerance : ± 100 ppm
Fragment Mass Tolerance: ± 0.5 Da
Max Missed Cleavages : 1
Instrument type : MALDI-TOF-TOF
Number of queries : 5

Mascot: http://www.matrixscience.com/

{MATRIX SCIENCE} Mascot Search Results

**User**	: **Unidad Proteomica CACTI**
**Email**	: **proteomica@uvigo.es**
**Search title**	: **Banda 4 MSMS**
**MS data file**	: **DATA.TXT**
**Database**	: **NCBInr 20070216 (4626804 sequences; 1596079197 residues)**
**Taxonomy**	: **Firmicutes (gram-positive bacteria) (320691 sequences)**
**Timestamp**	: **24 Feb 2011 at 16:20:48 GMT**
**Protein hits**	: gi|452163 alpha-toxin [Clostridium septicum]
	gi|30023346 uracil phosphoribosyltransferase [Bacillus cereus ATCC 14579]
	gi|15616326 uracil phosphoribosyltransferase [Bacillus halodurans C-125]
	gi|77683548 conserved hypothetical protein [Alkaliphilus metalliredigenes QYMF]
	gi|89099867 Aminopeptidase N [Bacillus sp. NRRL B-14911]

	NCBInr	Decoy	False discovery rate
Peptide matches above identity threshold	1	0	0.00 %
Peptide matches above homology or identity threshold	1	0	0.00 %

Mascot Score Histogram

Ions score is -10*Log(P), where P is the probability that the observed match is a random event.
Individual ions scores > 38 indicate identity or extensive homology (p<0.05).
Protein scores are derived from ions scores as a non-probabilistic basis for ranking protein hits.

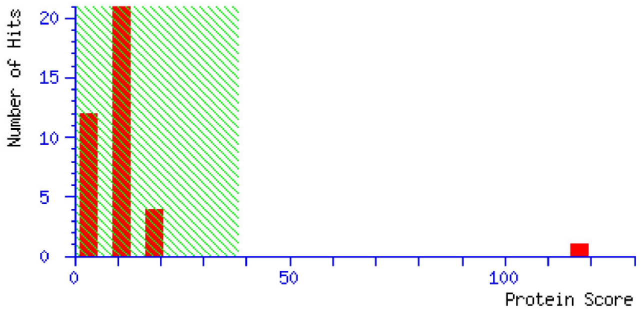

Peptide Summary Report

Format As | Peptide Summary | Help

Significance threshold p< 0.05 | Max. number of hits 5

Standard scoring ◉ MudPIT scoring ○ | Ions score or expect cut-off 0 | Show sub-sets 0

Show pop-ups ◉ Suppress pop-ups ○ | Sort unassigned Decreasing Score | Require bold red ☐

Overview Table

Click on column header to jump to entry in results list.
Move mouse over any indicator to highlight identical peptides.
Click on an indicator to see details of individual match.
Use check boxes to select sub-set of queries for new search.

Mouse over: -Query- | -Accession- | -Sequence-

Hit:	1	2	3	4	5
☑ 908.50 (1+)	●	●	●		●
☑ 1026.46 (1+)	●				
☑ 1130.59 (1+)	●			●	
☑ 1326.60 (1+)	●				

☑ 1416.60 (1+)

Select All | Select None | Search Selected | ☐ Error tolerant | Archive Report

1. gi|452163 **Mass:** 49926 **Score:** 117 **Matches:** 5(1) **Sequences:** 5(1)
alpha-toxin [Clostridium septicum]
☐ Check to include this hit in error tolerant search or archive report

Query	Observed	Mr(expt)	Mr(calc)	ppm	Miss	Score	Expect	Rank	Unique	Peptide
1	908.50	907.50	907.51	-17.57	0	17	9.7	1	U	K.VSYLLSAR.Y
2	1026.46	1025.46	1025.48	-21.50	0	18	4.2	1	U	K.NSYDALTSR.K
3	1130.59	1129.59	1129.61	-24.27	0	22	2.8	1	U	K.IIQDPEFIR.N
4	1326.60	1325.60	1325.62	-19.18	0	11	21	1	U	R.EDHTEDRPTVK.L
5	1416.60	1415.59	1415.61	-11.90	0	48	0.0037	1	U	R.YNPNDPYASGYR.A

Proteins matching the same set of peptides:
gi|27531076 **Mass:** 49634 **Score:** 117 **Matches:** 5(1) **Sequences:** 5(1)
alpha-toxin [Clostridium septicum]
gi|27531078 **Mass:** 49650 **Score:** 117 **Matches:** 5(1) **Sequences:** 5(1)
alpha-toxin [Clostridium septicum]
gi|27531080 **Mass:** 49588 **Score:** 117 **Matches:** 5(1) **Sequences:** 5(1)
alpha-toxin [Clostridium septicum]
gi|89257968 **Mass:** 49513 **Score:** 117 **Matches:** 5(1) **Sequences:** 5(1)
alpha-toxin [Clostridium septicum]

---

2. gi|30023346 **Mass:** 22943 **Score:** 16 **Matches:** 1(0) **Sequences:** 1(0)
uracil phosphoribosyltransferase [Bacillus cereus ATCC 14579]
☐ Check to include this hit in error tolerant search or archive report

Query	Observed	Mr(expt)	Mr(calc)	ppm	Miss	Score	Expect	Rank	Unique	Peptide
1	908.50	907.50	907.51	-17.57	1	16	13	2	U	K.ITYIRDK.N

Proteins matching the same set of peptides:
gi|47570682 **Mass:** 8607 **Score:** 16 **Matches:** 1(0) **Sequences:** 1(0)
uracil phosphoribosyltransferase [Bacillus cereus G9241]
gi|89203556 **Score:** 16 **Matches:** 1(0) **Sequences:** 1(0)
gi|89207647 **Score:** 16 **Matches:** 1(0) **Sequences:** 1(0)
gi|118480351 **Score:** 16 **Matches:** 1(0) **Sequences:** 1(0)

---

3. gi|15616326 **Score:** 16 **Matches:** 1(0) **Sequences:** 1(0)
uracil phosphoribosyltransferase [Bacillus halodurans C-125]
☐ Check to include this hit in error tolerant search or archive report

Query	Observed	Mr(expt)	Mr(calc)	ppm	Miss	Score	Expect	Rank	Unique	Peptide
1	908.50	907.50	907.51	-17.57	1	16	14	3	U	K.LTYIRDK.S

Proteins matching the same set of peptides:
gi|22096373 **Score:** 16 **Matches:** 1(0) **Sequences:** 1(0)
gi|23100439 **Score:** 16 **Matches:** 1(0) **Sequences:** 1(0)
gi|56421903 **Score:** 16 **Matches:** 1(0) **Sequences:** 1(0)

---

4. gi|77683548 **Score:** 15 **Matches:** 1(0) **Sequences:** 1(0)
conserved hypothetical protein [Alkaliphilus metalliredigenes QYMF]
☐ Check to include this hit in error tolerant search or archive report

Query	Observed	Mr(expt)	Mr(calc)	ppm	Miss	Score	Expect	Rank	Unique	Peptide
3	1130.59	1129.59	1129.61	-20.69	1	15	15	2	U	R.LIRSEEDLR.D

---

5. gi|89099867 **Score:** 15 **Matches:** 1(0) **Sequences:** 1(0)
Aminopeptidase N [Bacillus sp. NRRL B-14911]
☐ Check to include this hit in error tolerant search or archive report

Query	Observed	Mr(expt)	Mr(calc)	ppm	Miss	Score	Expect	Rank	Unique	Peptide
1	908.50	907.50	907.48	22.5	0	15	16	4	U	K.SVYIEAAR.A

Search Parameters

**Type of search**	**: MS/MS Ion Search**
**Enzyme**	**: Trypsin**
**Fixed modifications**	**: Carbamidomethyl (C)**
**Variable modifications**	**: Oxidation (M)**
**Mass values**	**: Monoisotopic**
**Protein Mass**	**: Unrestricted**
**Peptide Mass Tolerance**	**: ± 100 ppm**
**Fragment Mass Tolerance**	**: ± 0.5 Da**
**Max Missed Cleavages**	**: 1**
**Instrument type**	**: MALDI-TOF-TOF**
**Number of queries**	**: 5**

Mascot: http://www.matrixscience.com/
