# Supplementary material for: A simple electroelution method for rapid protein purification: isolation and antibody production of alpha toxin from Clostridium septicum
Source: PeerJ. 2017 Jun 22;5:e3407. doi: 10.7717/peerj.3407 (PMC5483040; doi:10.7717/peerj.3407)
Supplement: Supplemental Information 3 — Mascot Search Results of mass spectrometry identification for purified electroeluted protein. [file peerj-05-3407-s003.pdf]

Supplemental File 2: Mascot Search Results of mass spectrometry identification for purified electroeluted protein.

MATRIX SCIENCE Mascot Search Results

```
User          : Unidad Proteomica CACTI
Email         : proteomica@uvigo.es
Search title  : Banda 1 COOMASSIE_MSMS
MS data file  : DATA.TXT
Database      : NCBInr 20130503 (25052984 sequences; 8638475353 residues)
Taxonomy      : Firmicutes (gram-positive bacteria) (3326529 sequences)
Timestamp     : 7 May 2013 at 14:14:18 GMT
Protein hits  : gi|452163 alpha-toxin [Clostridium septicum]
```

	NCBInr	Decoy	False discovery rate
Peptide matches above identity threshold	2	0	0.00 %
Peptide matches above homology or identity threshold	2	0	0.00 %

Mascot Score Histogram

Ions score is -10*Log(P), where P is the probability that the observed match is a random event.
Individual ions scores > 49 indicate identity or extensive homology (p<0.05).
Protein scores are derived from ions scores as a non-probabilistic basis for ranking protein hits.

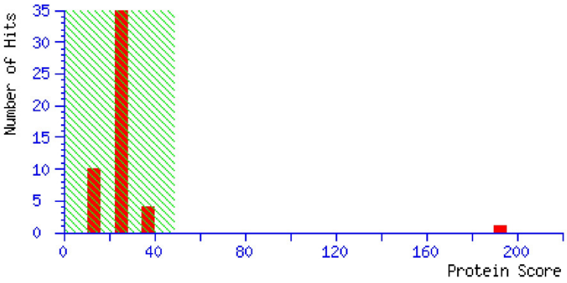

Peptide Summary Report

Format As [Peptide Summary] Help

Significance threshold p< [0.05] Max. number of hits [AUTO]

Standard scoring ◉ MudPIT scoring ○ Ions score or expect cut-off [0] Show sub-sets [0]

Show pop-ups ◉ Suppress pop-ups ○ Sort unassigned [Decreasing Score] Require bold red ☐

Preferred taxonomy [All entries]

Overview Table

Click on column header to jump to entry in results list.
Move mouse over any indicator to highlight identical peptides.
Click on an indicator to see details of individual match.
Use check boxes to select sub-set of queries for new search.

Mouse over: [-Query-]
[-Accession-] [-Sequence-]

Hit:	1
☑ 774.4174 (1+)	●
☑ 908.5189 (1+)	●
☑ 1130.6171 (1+)	●
☑ 1267.7096 (1+)	
☑ 1416.6077 (1+)	●
☑ 1759.9419 (1+)	

[Select All] [Select None] [Search Selected] ☐ **Error tolerant**

```
1.    gi|452163     Mass: 49926     Score: 193     Matches: 4(2)   Sequences: 4(2)
      alpha-toxin [Clostridium septicum]
    ☐ Check to include this hit in error tolerant search

      Query    Observed    Mr(expt)    Mr(calc)    ppm  Miss Score  Expect Rank Unique  Peptide
```

1	774.4174	773.4101	773.4112	-1.38	0	29	11	4	U	K.FGYLFK.N
2	908.5189	907.5116	907.5127	-1.22	0	16	1.2e+02	1	U	K.VSYLLSAR.Y
3	1130.6171	1129.6098	1129.6131	-2.94	0	56	0.012	1	U	K.IIQDPEFIR.N
5	1416.6077	1415.6004	1415.6106	-7.16	0	92	1.7e-06	1	U	R.YNPNDPYASGYR.A

Proteins matching the same set of peptides:

gi|27531076 **Mass:** 49634 **Score:** 193 **Matches:** 4(2) **Sequences:** 4(2)
alpha-toxin [Clostridium septicum]

gi|27531078 **Mass:** 49650 **Score:** 193 **Matches:** 4(2) **Sequences:** 4(2)
alpha-toxin [Clostridium septicum]

gi|27531080 **Mass:** 49588 **Score:** 193 **Matches:** 4(2) **Sequences:** 4(2)
alpha-toxin [Clostridium septicum]

gi|89257968 **Mass:** 49513 **Score:** 193 **Matches:** 4(2) **Sequences:** 4(2)
alpha-toxin [Clostridium septicum]

gi|169639783 **Mass:** 49997 **Score:** 193 **Matches:** 4(2) **Sequences:** 4(2)
alpha-toxin [Clostridium septicum]

gi|169639785 **Mass:** 49997 **Score:** 193 **Matches:** 4(2) **Sequences:** 4(2)
alpha-toxin [Clostridium septicum]

gi|169639787 **Mass:** 49990 **Score:** 193 **Matches:** 4(2) **Sequences:** 4(2)
alpha-toxin [Clostridium septicum]

gi|169639791 **Mass:** 49994 **Score:** 193 **Matches:** 4(2) **Sequences:** 4(2)
alpha-toxin [Clostridium septicum]

gi|169639793 **Mass:** 50029 **Score:** 193 **Matches:** 4(2) **Sequences:** 4(2)
alpha-toxin [Clostridium septicum]

gi|169639797 **Mass:** 49924 **Score:** 193 **Matches:** 4(2) **Sequences:** 4(2)
alpha-toxin [Clostridium septicum]

gi|169639799 **Mass:** 49897 **Score:** 193 **Matches:** 4(2) **Sequences:** 4(2)
alpha-toxin [Clostridium septicum]

gi|169639801 **Mass:** 49925 **Score:** 193 **Matches:** 4(2) **Sequences:** 4(2)
alpha-toxin [Clostridium septicum]

gi|313585702 **Mass:** 49575 **Score:** 193 **Matches:** 4(2) **Sequences:** 4(2)
alpha-toxin [Clostridium septicum]

Peptide matches not assigned to protein hits: (no details means no match)

	Query	Observed	Mr(expt)	Mr(calc)	ppm	Miss	Score	Expect	Rank	Unique	Peptide
☑	1	774.4174	773.4101	773.3782	41.3	0	31	5.5	1		MGYIFK + Oxidation (M)
☑	4	1267.7096	1266.7023	1266.6642	30.1	1	27	8.5	1		YGEMLKELIR + Oxidation (M)
☑	6	1759.9419	1758.9346	1758.8900	25.3	1	13	1.6e+02	1		ASYITGTTYNIAGGKSR

Search Parameters

Type of search : MS/MS Ion Search
Enzyme : Trypsin
Fixed modifications : Carbamidomethyl (C)
Variable modifications : Oxidation (M)
Mass values : Monoisotopic
Protein Mass : Unrestricted
Peptide Mass Tolerance : ± 100 ppm
Fragment Mass Tolerance: ± 0.5 Da
Max Missed Cleavages : 1
Instrument type : MALDI-TOF-TOF
Number of queries : 6

Mascot: http://www.matrixscience.com/
